# Acute Reversible Atypical Takotsubo Cardiomyopathy Following Trastuzumab and 5-Fluorouracil in a Young Patient

**DOI:** 10.7759/cureus.72764

**Published:** 2024-10-31

**Authors:** Christos Lafaras, Kyranna Lafara, Thomas Achladas, Vassiliki Koukoulitsa, Evdokia M Mandala

**Affiliations:** 1 Department of Cardiology-Oncology, Theagenio Cancer Hospital, Thessaloniki, GRC; 2 Forth Department of Medicine, School of Medicine, Hippokration General Hospital, Aristotle University of Thessaloniki, Thessaloniki, GRC

**Keywords:** 5-fluorouracil, acute cardiac dysfunction, cardiotoxicity, gastroesophageal junction cancer, trastuzumab

## Abstract

Combined therapeutic regimens, including molecular targeted agents, are considered standard treatment for advanced gastric and gastroesophageal junction cancer. We present an extremely rare case of acute reversible cardiac dysfunction in a 20-year-old patient after administration of trastuzumab plus 5-fluorouracil for the treatment of metastatic gastroesophageal junction cancer. During the first day of chemotherapy, the patient complained of retrosternal pain radiating to the scapular regions bilaterally. Infusion was immediately stopped, and signs of myocardial ischemia were depicted on the electrocardiogram (ECG) with a positive high-sensitivity troponin T assay, confirming the diagnosis of acute coronary syndrome. Echocardiography revealed global left ventricular hypokinesia and severe systolic dysfunction. After appropriate treatment, the patient recovered, and three days later, he had preserved left ventricular function. Coronary angiography ruled out coronary artery disease. The reported case of acute and severe reversible cardiac dysfunction due to a specific combination regimen in a patient with low pre-treatment risk of cancer therapy-related cardiovascular toxicity (CTR-CVT) highlights the gaps in evidence regarding the risk assessment tools for Heart Failure Association-International Cardio-Oncology Society (HFA-ICOS), representing an atypical variant of takotsubo cardiomyopathy. This case underscores the importance of developing personalized surveillance protocols during therapeutic interventions to mitigate potential adverse effects.

## Introduction

Gastric and gastroesophageal junction cancer is the fifth leading cause of cancer and the second leading cause of cancer-related mortality worldwide. Despite the overall decline in gastric cancer, there has been a dramatic increase in the incidence of gastric-cardiac cancer due to environmental factors or chemical carcinogens. Additionally, there has been an increase in the incidence of gastric cancer among young adults, possibly due to autoimmune gastritis and dysbiosis secondary to the increased use of antibiotics and acid suppressants. Although there has been a steady decline in gastric cancer mortality, rates remain high because most patients present with advanced disease. Approximately 15-20% of advanced gastric and gastroesophageal junction adenocarcinomas exhibit overexpression or amplification of the human epidermal growth factor receptor 2 (HER2) [1]. In addition to traditional chemotherapeutic regimens, molecular targeted agents are evolving as new treatment strategies for improving outcomes in advanced gastric and gastroesophageal junction cancer [2]. The HER2 receptor is a member of the human epidermal growth factor receptor (HER) family, which includes epidermal growth factor receptor (EGFR) (HER1), HER3, and HER4. The structure of the HER-2 receptor consists of four extracellular domains, one transmembrane domain, and a cytoplasmic tyrosine kinase domain. This structural composition is crucial for its function in cell signaling pathways that regulate cell growth and differentiation. The receptor can form homodimers without the need for ligand binding leading to the activation of its intrinsic kinase activity and resulting in downstream signaling that promotes cell proliferation and survival. HER2 can also participate in ligand-dependent heterodimerization with other members of the HER family. The co-expression of HER2 and HER3 leads to high-affinity binding of heregulin and subsequent tyrosine phosphorylation events. This interaction results in one of the most potent mitogenic signals within this receptor family. The signaling cascade initiated by the activation of the HER2/HER3 heterodimer via the mitogen-activated protein kinase (MAPK) pathway and the phosphatidylinositol 3-kinase (PI3K) pathway promotes migration, cell growth, adhesion, and differentiation, and inhibits apoptosis [3].

Human epidermal growth factor receptor 2 (HER2) is a transmembrane tyrosine kinase receptor involved in many normal cellular processes, affecting the regulation of cell growth and survival. Trastuzumab is a fully-humanized monoclonal antibody, an antagonist of the HER2 receptor, and is indicated for the treatment of HER2-overexpressing breast cancer (both adjuvant and metastatic) and metastatic gastric or gastroesophageal junction adenocarcinoma [3,4]. It exerts its action via a combination of antibody-dependent cytotoxicity, reduced shedding of the extracellular domain, inhibition of dimerization, and possibly receptor downregulation [4]. Apart from common adverse reactions observed with most chemotherapeutic agents, life-threatening complications associated with trastuzumab administration include cardiomyopathy, infusion reactions, embryo-fetal toxicity, pulmonary toxicity, and exacerbation of chemotherapy-induced neutropenia. Cardiovascular complications vary from an asymptomatic decline in left ventricular ejection fraction (LVEF) to severe left ventricular cardiac dysfunction, arrhythmias, hypertension, disabling cardiac failure, cardiomyopathy, and cardiac death. There is a 4-6-fold increase in the incidence of symptomatic myocardial dysfunction among patients receiving trastuzumab, either as single or combination therapy [5,6]. Regarding 5-fluorouracil (5-FU), cardiotoxicity may manifest as myocardial ischemia with anginal pain and/or electrocardiographic changes (elevation or depression of the ST segment, inverted T wave), heart failure (impaired ventricular contractility), and arrhythmia [7]. Clinical features of cardiotoxicity due to combined chemotherapy with both trastuzumab and 5-fluorouracil in low-risk patients, according to cardiovascular toxicity risk stratification models before anticancer therapy, are not extensively studied.

## Case presentation

A 20-year-old male patient suffering from dysphagia (mainly from solid foods) and weight loss (20% of body weight in the past three months) was referred to our department for further evaluation and management. He was a smoker (four pack-years) with a non-remarkable personal and family medical history, no known psychiatric illness, and mild psychological distress due to the anticipation of the disease's diagnosis. His vital signs on admission were within normal limits (blood pressure {BP}=120/80 mmHg, heart rate {HR}=80 bpm, oxygen saturation {SpO_2_}=99%).

One month before referral, he had undergone radiological evaluation with barium esophagography and computed tomography (CT) of the thorax, upper and lower abdomen, which revealed stenosis of the lower third of the esophagus and the gastroesophageal junction, as well as multiple ulcerative lesions of the gastroesophageal junction. The esophagogastroscopy confirmed the stenosis, diagnosed a diaphragmatic hernia, and identified multiple flame-like gastric mucosa islets in the gastroesophageal junction. Biopsies from the region were negative for malignancy. Due to persistent symptoms, the patient was referred to the gastroenterology department of our hospital, where he underwent three sessions of diagnostic-therapeutic endoscopy. Barrett’s esophagus, gastroesophageal reflux disease, and diaphragmatic hernia were diagnosed, along with gastroesophageal junction stenosis treated with balloon dilations. Immunohistopathological findings revealed a poorly differentiated adenocarcinoma of the gastroesophageal junction with multiple sites of signet ring cells and positive HercepTest (overexpression of HER2). For staging purposes, new thoracic and abdominal CTs were requested, which depicted a soft-tissue mass at the gastroesophageal junction (maximum diameter=31.8 mm) and multiple enhancing low-density hepatic lesions (the largest in segment IVa, diameter=4.6 mm), characteristic of metastatic disease. A subsequent MRI confirmed these findings.

A stage IV gastroesophageal junction adenocarcinoma was diagnosed, and the patient was referred to our department for further management. He had a performance status (PS) of zero, all laboratory tests were within normal limits, the pre-chemotherapy cardiovascular evaluation showed no abnormalities, and tumor markers were CEA=9.58 ng/mL and CA19-9=540.32 U/mL. Standard first-line systemic therapy was decided. Considering the patient’s young age and performance status, and according to the National Comprehensive Cancer Network (NCCN) guidelines, we chose a combination of docetaxel, cisplatin, and fluorouracil (DCF) and trastuzumab every 21 days [8].

**Figure 1:**
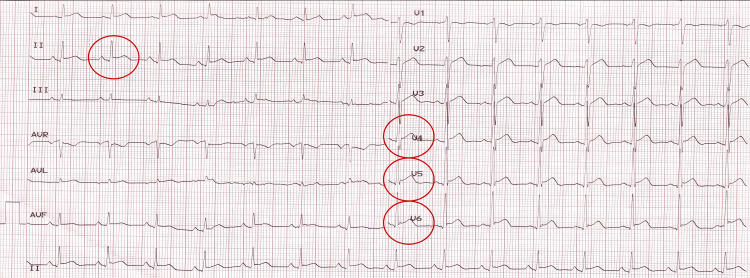
ECG of the patient during the acute phase with elevated ST segments in leads II, V4, V5, V6 indicative of an acute coronary syndrome.

During the first day of chemotherapy, after the administration of trastuzumab (8 mg/kg loading dose) and during the first minutes of continuous 5-FU infusion, the patient complained of intermittent precordial chest pain radiating to the scapular regions bilaterally, numbness in the left arm, epigastralgia, nausea, headache, and arthralgia. The 5-FU infusion was stopped, and signs of myocardial ischemia (initially negative T waves in leads II, V4, V5, V6) were observed on the electrocardiogram (ECG), with a positive high-sensitivity troponin T assay (hsTnT=70.84 ng/L; normal limits=0-14 ng/L) confirming the diagnosis. The patient was transferred to the cardiology department, where the ischemic episode was evolving with an ECG pattern of acute coronary syndrome (elevated ST segment in leads II, V4, V5, V6) (Figure 1).

**Figure 2:**
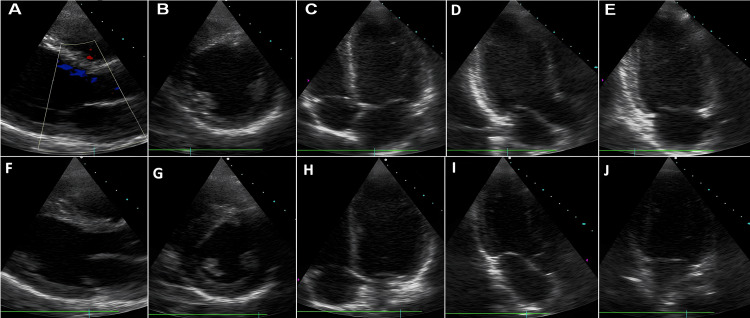
Two-dimensional echocardiographic images in the acute phase (upper panel) and after recovery (lower panel). Generalized severe hypokinesia, dilatation of the left ventricle, and low LVEF in the acute phase were the major findings (A-E). Three days after the episode, the heart was fully recovered (F-J). A and F show parasternal axis, B and G show short axis, C and H show apical four-chamber view, D and I show apical three-chamber view, and E and J show apical two-chamber. LVEF: left ventricular ejection fraction

**Figure 3:**
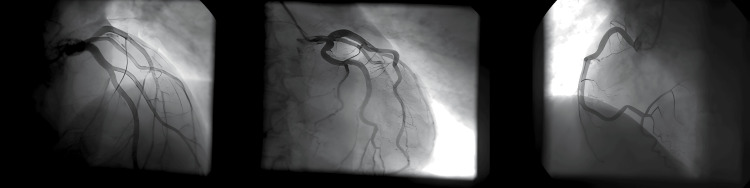
Coronary angiogram with normal coronary vessels.

The echocardiographic study in the acute phase demonstrated a significant decline in the left ventricular ejection fraction (LVEF=27%), generalized severe hypokinesia, and dilatation of the left ventricle. The patient was admitted to the intensive care unit (ICU) for further support, where he received carvedilol, ramipril, furosemide, clopidogrel, aspirin, and low molecular weight heparin. Three days after the acute episode, a complete recovery of cardiac function was recorded with an LVEF of 62%, compatible with acute ischemic reversible cardiac dysfunction (Figures 2A-2J). He eventually underwent a coronary angiogram, which depicted no critical stenoses of the coronary vessels (Figure 3).

The chemotherapy was discontinued for 15 days. After a completely normal cardiovascular re-evaluation, a new regimen with docetaxel monotherapy (75 mg/m^2^ every 21 days) was initiated. After six cycles of chemotherapy, the patient remained in PS=0, with no further toxicity complications. Tumor markers were CEA=3.18 ng/mL and CA19-9=16.35 U/mL, with a weight gain of 15 kg and a decrease in the number and size of the hepatic metastatic lesions.

## Discussion

HER2 overexpression is variably noted in gastroesophageal junction adenocarcinoma from 0% to 43% [9]. Trastuzumab plus chemotherapy is now a standard first-line treatment option for patients with advanced HER2-positive gastroesophageal cancer, significantly improving response rate, median progression-free survival, and median overall survival [3,8,9]. The patient underwent a combined chemotherapeutic regimen according to the recent NCCN guidelines for the treatment of gastroesophageal junction cancer.

Clinical evidence of myocardial dysfunction or significant decline in LVEF had an incidence of 0.4-28% in trastuzumab breast cancer studies, most often manifesting with asymptomatic reduction of LVEF and less frequently with clinical signs of heart failure and arrhythmias. The incidence is higher in patients receiving concurrent trastuzumab and anthracycline and cyclophosphamide (AC) (27%) and lower in patients receiving trastuzumab plus paclitaxel (13%) or trastuzumab alone (3-7%). Chest wall irradiation, antihypertensive therapy, and preexisting cardiac dysfunction are additional risk factors, along with obesity and age over 60 years [2,10-12]. While cardiac dysfunction is reversible in most patients, in rare cases the drug can trigger severe congestive heart failure with progression to death, formation of intramural thrombus, or even mimic acute coronary syndrome with complete left bundle branch blockade [2,10-12].

The exact mechanism of trastuzumab cardiotoxicity remains unknown. Several hypotheses have been suggested and are under investigation, such as the antibody-directed cell cytotoxic activity of trastuzumab, imbalance in neuregulin homeostasis, induction of mitochondrial dysfunction, defective HER2 signaling, signaling for the opening of calcium channels, and microRNA (miRNA) dysregulation. The antibody-directed cell cytotoxic activity of trastuzumab, although an obvious mode of action, has not been proven adequate to cause clinically relevant cardiotoxicity, particularly in patients with overexpression of the HER2 receptor in myocardial cells [2,13,14]. Trastuzumab is believed to upregulate angiotensin II, leading to an increase in ROS production and inhibition of neuregulin signaling. Calcium channels in cardiomyocytes are important for maintaining cardiac contractility, and the inhibition of HER2 signaling, with subsequent changes in the expression of BCL-X proteins and adenosine triphosphate (ATP) depletion, causes contractile dysfunction (functional effect) without any changes in cellular structure (morphological effect) [10].

The incidence of 5-FU cardiotoxicity ranges from 1.2% to 18% and may manifest as angina, myocardial infarction, coronary dissection, prolonged QT interval, supraventricular tachycardia, ventricular tachycardia, heart failure (about 3.5%), myopericarditis, cardiomyopathy, cardiogenic shock, and sudden death [7,14]. Shorter bolus regimens are safer (cardiotoxicity incidence between 1.6% and 3%), while more prolonged continuous infusions increase the cardiotoxicity incidence to 7.6-18%. Spasms of a coronary artery, autoimmune-mediated injury of the myocardium, endothelial damage, thrombogenic effects or thrombus formation, direct myocardial toxicity causing necrosis, global myocardial dysfunction, and the accumulation of toxic metabolites are implicated to varying degrees and possibly in a synergistic mode of action [14]. Elevated cardiac enzymes and NT-proBNP levels may accompany the underlying evolving pathophysiological mechanism. Symptoms tend to occur most commonly during the first cycle of administration, 3-48 hours after initiation of the infusion, and may last up to 12 hours after cessation, even in patients without a prior history of heart disease, as in our patient [14].

In the present case, the patient was a 20-year-old young adult receiving chemotherapy for the first time and subsequently indicated as low risk for cardiac toxicity. However, a pre-treatment cardiac evaluation with ECG and echocardiography was performed because of the high cardiotoxic regimen (loading dose of trastuzumab and four days continuous infusion of 5-FU) that would be applied. During the initiation of the treatment, he displayed symptoms indicative of acute coronary syndrome with ECG changes and elevation of cardiac enzymes. Echocardiographic findings were consistent with acute heart failure, with a dramatic decrease in LVEF that was reversed three days later. To the best of our knowledge, this is a unique case of acute reversible cardiac dysfunction in a patient treated with this combined chemotherapy regimen (trastuzumab plus 5-FU). Although sporadic case reports of acute decompensated heart failure syndrome are described in the literature, all include older patients with prior chemotherapy with anthracyclines and a longer recovery period [10,12]. Trastuzumab cardiotoxicity occurs more frequently in patients who have overexpression of HER2 receptors in myocardial cells. However, its antiangiogenic effects are mediated through HER3 and HER4 receptors as well, which are responsible for possible enhanced and/or individualized cardiotoxicity. Stress cardiomyopathy, also known as takotsubo cardiomyopathy, is a rapidly reversible form of acute heart failure classically triggered by stressful events. It is associated with a distinctive left ventricular contraction pattern described as apical akinesis/ballooning with hyperdynamic contraction of the basal segments in the absence of obstructive coronary artery disease [15,16]. The mean age of patients presenting with this syndrome is approximately 62-75 years [15-17]. In a case series, approximately 85-100% of patients diagnosed with takotsubo were women. Although the pathogenesis is not well understood, postulated mechanisms include catecholamine excess (similar reversible cardiomyopathy with global or focal dysfunction in patients with рhеοϲhrοmοϲytomа), microvascular dysfunction, and coronary artery spasm, resulting in myocardial stunning. Cardiac and systemic inflammation has also been reported, but it is unknown if this is a nonspecific consequence of the myocardial injury or a pathophysiologic mechanism. There have been reports of familial cases, raising the possibility of a genetic predisposition (genetic heterogeneity with possible polygenic basis). Additionally, patients with psychiatric or neurologic disorders may be predisposed to develop stress cardiomyopathy. Studies of patients with stress cardiomyopathy suggest hypoconnectivity between central brain regions associated with autonomic functions and regulation of the limbic system compared with healthy controls. The incidence of stress cardiomyopathy among individuals exposed to physical or emotional stress is not known, as it requires validation in larger series, but it appears that this phenomenon is not uncommon in a medical intensive care unit population. Chemotherapy-induced takotsubo cardiomyopathy is a relatively new phenomenon, as the literature increasingly suggests an association between cancer, chemotherapeutic drugs, and stress cardiomyopathy [16-18]. On the contrary, our case is a young male patient with mild emotional stress who developed atypical cardiomyopathy out of the intensive care unit. The more generalized global involvement of the heart may represent an atypical variant of chemotherapy-induced "broken-heart syndrome." Several algorithms can be implemented to minimize cardiac toxicity, including careful pre-treatment cardiovascular evaluation to identify high-risk patients, regular cardiovascular monitoring during treatment to recognize early cardiac toxicity, and effective measures when cardiac dysfunction is detected [19,20]. Additionally, genetic factors play a significant role in determining individual susceptibility to cardiovascular damage from anticancer drug exposure. Thus, genetic screening may offer a valuable tool for identifying patients at higher risk of developing cancer therapy-related cardiovascular toxicity (CTR-CVT) and allow for personalized interventions that can reduce the incidence and severity of cardiotoxicity. By incorporating genetic information into clinical decision-making processes, healthcare providers can optimize cancer treatment strategies and improve patient safety and outcomes.

## Conclusions

Candidate patients receiving combined treatment (chemotherapy plus targeted therapy) should be monitored closely during treatment, regardless of preexisting heart disease. Early detection and treatment of myocardial dysfunction are crucial in these patients. Angiotensin-converting enzyme (ACE) inhibitors and/or beta-blockers should be recommended to achieve potential cardioprotective benefits. Cancer treatment is a matter of urgency, but cardiac side effects may be life-threatening conditions. Developing personalized surveillance protocols during therapeutic interventions is crucial for mitigating potential adverse effects in patients undergoing cancer treatment. Even those with low pre-treatment risk require careful monitoring to prevent or manage cancer therapy-related cardiovascular toxicity (CTR-CVT) effectively.
